# Erfolgreiche multimodale Therapie der CD30‐positiven follikulotropen Mycosis fungoides

**DOI:** 10.1111/ddg.70172

**Published:** 2026-08-04

**Authors:** Chiara L. Blomen, Inga Hansen‐Abeck, Andrea Baehr, Stefan W. Schneider, Christoffer Gebhardt, Nina Booken

**Affiliations:** ^1^ Klinik und Poliklinik für Dermatologie und Venerologie Universitätsklinikum Hamburg‐Eppendorf Hamburg Deutschland; ^2^ Klinik für Strahlentherapie und Radioonkologie Universitätsklinikum Hamburg‐Eppendorf Hamburg Deutschland

Sehr geehrte Herausgeber,

 die follikulotrope Mycosis fungoides (FMF) ist eine seltene und eigenständige Variante der Mycosis fungoides mit perifollikulärer Infiltration CD4‐positiver T‐Zellen, begleitet von follikulärer Muzinose bei meist fehlender Epidermotropie. Es werden ein frühes und ein fortgeschrittenes Krankheitsstadium unterschieden, die sich in ihrer klinischen Präsentation und Prognose unterscheiden.^1^ Die FMF tritt typischerweise im Kopf‐Hals‐Bereich und am Rumpf auf. Klinisch imponieren in der Frühphase erythematöse *Patches*, die von Alopezie, akneiformen Exanthemen einschließlich Komedonen sowie Milien oder Keratosis‐pilaris‐ähnlichen Läsionen begleitet sein können.^2^ Im fortgeschrittenen Stadium bestehen Plaques oder Tumoren, analog zur klassischen Mycosis fungoides mit schlechterer Prognose.^1^

Bei CD30‐Positivität ist die Abgrenzung zu der lymphomatoiden Papulose (LyP) und dem primär kutanen CD30‐positiven großzelligen anaplastischen T‐Zell‐Lymphom (cALCL) relevant und nur durch klinisch‐pathologische Korrelation möglich.^3^ Die LyP ist klinisch durch vollständige Abheilung der papulonodulären Läsionen binnen weniger Wochen gekennzeichnet.^4^ Das cALCL geht mit solitären, häufig ulzerierten Läsionen einher, während die FMF durch ein Nebeneinander von *Patches*, Plaques und gegebenenfalls Tumoren gekennzeichnet ist.

Die Therapie der FMF ist abhängig von dem Erkrankungsstadium und kann herausfordernd sein,^2^ weshalb insbesondere synergistisch wirksame, multimodale Therapiekonzepte berücksichtigt werden sollten. Neben topischen, UV‐ und strahlentherapeutischen Ansätzen können systemische Erstlinientherapien wie Bexaroten, Interferon‐alpha und Methotrexat oder in der Zweitlinientherapie Brentuximab vedotin (BV) und Mogamulizumab eingesetzt werden^5^, ^6^, ^7^. BV ist ein Antikörper gegen CD30, der mit dem antimikrotubulären Wirkstoff Monomethyl‐Auristatin E (MMAE) konjugiert ist. Nach Bindung an die CD30‐positive T‐Zelle und Internalisierung entfaltet MMAE seine apoptotische Wirkung über die Zerstörung der Mikrotubuli. BV wirkt durch die Erhöhung von DNA‐Doppelstrangbrüchen als Radiosensibilisator in Tumorzellen.^8^, ^9^ Nach der Kombination aus ultra‐hypofraktionierter niedrig‐dosierter Ganzhautbestrahlung und Brentuximab vedotin^9^ wurden nahezu vollständige Abheilungen der Hautläsionen des fortgeschrittenen kutanen T‐Zell‐Lymphoms beschrieben.

Ein 64‐jähriger Patient stellte sich aufgrund eines seit einem Jahr exophytisch wachsenden Tumors am Capillitium vor, begleitet von erythematösen Plaques und einem akneiformen Exanthem am Rumpf (Abbildung 1). Weitere Beschwerden wurden ebenso wie regelmäßige Medikamenteneinnahme verneint.

**ABBILDUNG 1:**
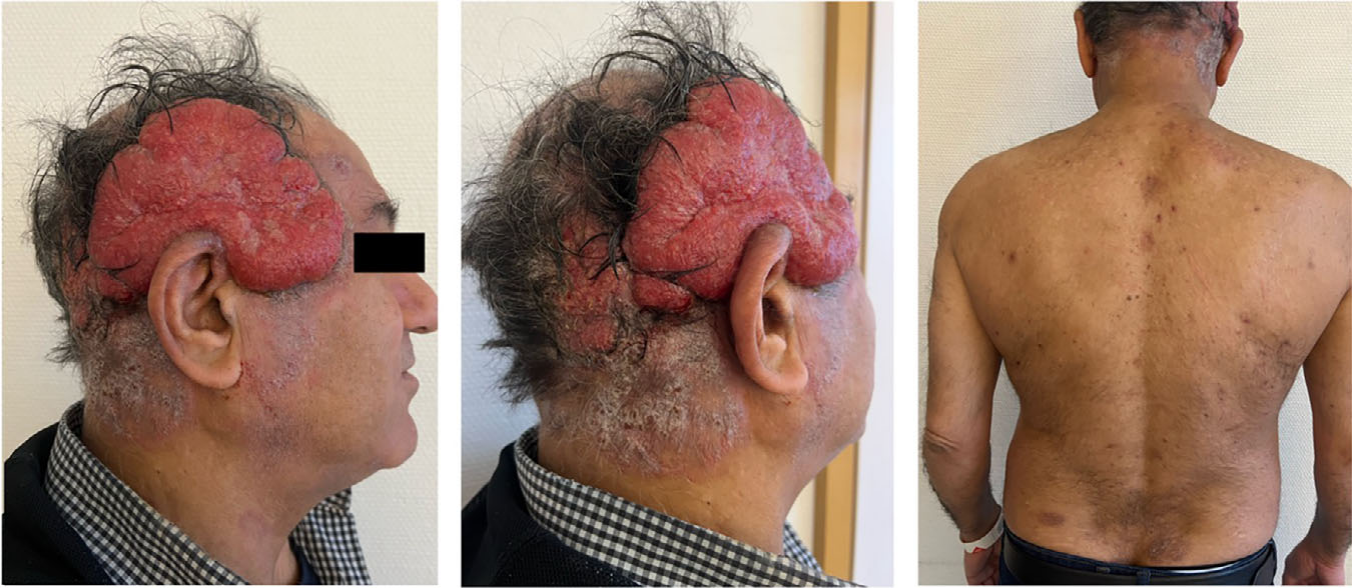
Initiale Präsentation: Tumor am Capillitium und Plaques sowie akneiformes Exanthem am Rumpf.

Hautbiopsate zeigten eine pleomorphe, follikulotrope T‐Zell‐Proliferation mit CD30‐positiver großzelliger Transformation. Im Plaque‐Anteil kamen 15–20% und im nodulären Anteil 40% CD30‐positive T‐Zellen zur Darstellung. Aufgrund einer zervikalen Lymphadenopathie erfolgte eine Lymphknotenbiopsie, mittels derer eine nodale Beteiligung ausgeschlossen werden konnte. Weitere Untersuchungen konnten eine Blut‐ und Organbeteiligung (mittels FACS‐Analyse, Computertomografie von Hals bis Becken und Magnetresonanztomografie des Kopfes) ausschließen. In klinisch‐pathologischer Korrelation wurde die Diagnose einer CD30‐positiven FMF (Tumorklassifikation nach ISCL/EORTC T3 N1a M0 B0, Stadium IIB) gestellt.

Gemäß des interdisziplinären Tumorboards wurde zunächst Methotrexat 15 mg subkutan und Folsäure einmal wöchentlich über drei Wochen sowie eine antibiotische Therapie in Anlehnung an das sogenannte Lindahl‐Schema durchgeführt.^10^ Die Rationale hinter einer Antibiotika‐Therapie bei kutanen T‐Zell‐Lymphomen besteht in der Tumor‐assoziierten Hautbarrierestörung und der damit assoziierten verstärkten *Staphylococcus aureus* Besiedlung. Diese führt nicht nur zu vermehrten Bakteriämien und damit erhöhter Morbidität und Mortalität, sondern auch zu gesteigerter Freisetzung von Enterotoxinen. Diese fungieren als Superantigene für T‐Zellen und können zu Tumorprogress kutaner T‐Zell‐Lymphome beitragen.^10^

Bei unzureichendem Therapieansprechen und gastrointestinalen Nebenwirkungen unter Methotrexat und hoher CD30‐Positivität wurde nach drei Wochen als Zweitlinientherapie auf eine Kombinationstherapie aus BV in Standarddosis (1,8 mg/kgKG) und ultra‐hypofraktionierter lokaler Radiatio^11^ umgestellt. Bereits nach zwei Zyklen BV und Applikation von lokaler Bestrahlung am Capillitium mittels 8 Gy in zwei Fraktionen zu je 4 Gy zeigte sich eine signifikante Verbesserung des klinischen Bildes. Unter der Therapie mit BV trat nach fünf Zyklen eine periphere Polyneuropathie (PNP) Grad II nach CTCAE‐Kriterien auf, weshalb die Dosierung auf 1,2 mg/kgKG reduziert wurde. Nach insgesamt sieben BV‐Zyklen erfolgte eine Exzision des residuellen Tumors mit Nachweis von ausgedehnten entzündlichen narbigen Veränderungen und Nachweis lediglich vereinzelter residueller Lymphomzellen. Anschließend erfolgte eine Spalthauttransplantation zur Defektdeckung und zur Erhaltungstherapie wurde Bexaroten 150 mg/m^2^ pro Tag verabreicht (Abbildung 2). Hierunter zeigte sich nach sechs Monaten kein Hinweis auf ein Rezidiv am Capillitium bei residuellen kutanen Manifestationen am Rumpf.

**ABBILDUNG 2:**
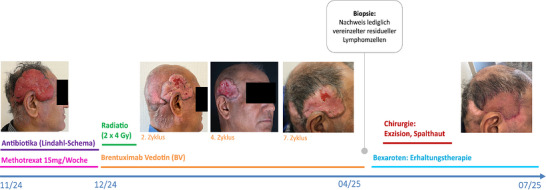
Zeitlicher Verlauf der Therapie und des klinischen Ansprechens.

Dieser Fall unterstreicht, dass die CD30‐positive FMF differenzialdiagnostisch sorgfältig von dem cALCL und der LyP abgegrenzt werden muss. Die korrekte Diagnosestellung gelingt durch klinisch‐pathologische Korrelation. Die Therapie der FMF ist abhängig von dem Erkrankungsstadium. Die Kombination aus BV und ultra‐hypofraktionierter Radiatio kann insbesondere bei hoher CD30‐Expression zu exzellenten klinischen Ergebnissen führen und eine signifikante Reduktion des Tumorvolumens ermöglichen.

## DANKSAGUNG

Open access Veröffentlichung ermöglicht und organisiert durch Projekt DEAL.

## INTERESSENKONFLIKT

Nina Booken erhielt Honorare von Takeda GmbH & Co. KG.
